# Impacts of Left Atrial Appendage Treatments on Mitral Valve Diseases during Surgical Ablations

**DOI:** 10.31083/j.rcm2501013

**Published:** 2024-01-09

**Authors:** Can Zhou, Yichen Zhao, Cheng Zhao, Qing Ye, Jianzeng Dong, Jiangang Wang

**Affiliations:** ^1^Department of Cardiology, Beijing Anzhen Hospital, Capital Medical University, 100000 Beijing, China; ^2^Department of Cardiac Surgery, Beijing Anzhen Hospital, Capital Medical University, 100000 Beijing, China

**Keywords:** atrial fibrillation, left atrial appendage, left atrial function, surgical ablation, atrial functional mitral regurgitation

## Abstract

**Background:**

Left atrial appendages (LAAs) play an important role in regulating left atrial function, and much evidence supports the possibility that changes in left atrial structure may cause or worsen mitral regurgitation. This study intended to investigate the outcomes of patients with mitral regurgitation who underwent left atrial appendage closure (resection or endocardial closure) during isolated surgical ablations.

**Methods:**

Patients with mild or moderate mitral regurgitation who received isolated surgical ablations for atrial fibrillation (AF) in our center from 2013 to 2022 were referred. During follow-up, each clinical visit was composed of medical interrogation, a 24 h Holter, and echocardiographic evaluation. Death, atrial fibrillation, worsening of mitral regurgitation, and stroke were evaluated as outcomes. Freedom from outcomes whose results were adjusted by inverse probability of treatment weighting for causal effects after acquiring propensity scores.

**Results:**

A total of 456 patients were enrolled in this study. During a median follow-up of 48 months, 30 deaths and 11 cases of stroke were observed. After adjustments, no significant differences in terms of death or stroke were observed among the three groups. Patients who underwent resection or endocardial closure during surgical ablations had a higher risk of mitral regurgitation worsening during follow-up (*p* < 0.05). During the whole follow-up, patients who underwent left atrial appendage interventions showed significantly larger left atrial and mitral annular diameters, as well as lower tethering height than those who had left atrial appendage preserved (all *p* < 0.05).

**Conclusions:**

Mitral regurgitation was more likely to get worse when patients with fundamental mitral diseases underwent LAA interventions during isolated surgical AF ablations. In the absence of LAA, the dilation of the left atrium and mitral annulus may ultimately lead to worsening of regurgitation.

## 1. Introduction

Left atrial appendage (LAA) is highly associated with the formation of left atrial thrombosis and atrial fibrillation (AF) [1, 2, 3]. Surgical LAA intervention, including LAA ligation and LAA excision, has been a common treatment for AF when patients undergo cardiac surgery [4, 5]. While some evidence supported that LAA resection or endocardial closure may cause the loss of left atrial physiological functions, like reservoir and contractile functions [6, 7, 8]. Some evidence also showed that the left atrial pressure and size increased after LAA was excised or excluded [9, 10], which revealed that a potential relationship may be present between LAA and LA remodeling. It is well known that atrial remodeling could contribute to functional mitral regurgitation (MR) [11, 12], and sometimes may lead to the worsening of MR. This study focused on patients with MR diseases who underwent surgical ablation, trying to illustrate whether LAA interventions could affect the outcomes of patients with fundamental MR diseases.

## 2. Materials and Methods

### 2.1 Patient Enrollment

Patients with mild or moderate degenerative MR who received isolated surgical ablations for paroxysmal or persistent AF diagnosed on a 12-lead electrocardiogram in our center from 2013 to 2022 were referred. The exclusion criteria were as follows: (1) patients with organic valvular diseases who needed invasive interventions according to the guidelines; (2) patients with mechanical or biological prosthesis; (3) patients who received any other cardiac surgeries during ablations; (4) other etiologies of mitral valve diseases like rheumatic mitral valve diseases and secondary mitral valve regurgitation; (5) permanent pacemaker implantations. Patients were grouped according to the treatment methods for LAA (LAA resection, endocardial closure or preservation)

### 2.2 Transthoracic Echocardiography

Two-dimensional echocardiography and doppler color flow imaging (IE33; Philips Medical Systems, Andover, MA, USA) were performed on all patients. Despite the routine geometric examination of left heart (left atrium and left ventricle). MR and tricuspid regurgitation (TR) grade were assessed by a multiparametric approach [13], including qualitative, semi-quantitative, and quantitative parameters, which was graded from 0 to 4, where 0, none or trivial; 1+, mild; 2+, moderate; 3+, moderate to severe; and 4+, severe. Mitral annular diameter (MAD) was acquired from a four-chamber apical view at the end of expiration. The measurement of the mitral tethering height (TH) was done by calculating the distance from the mitral annular plane to the leaflet coaptation point orthogonally.

### 2.3 Surgical Procedure 

Ablation was carried out with bipolar Cardioablate (Medtronic, Minneapolis, MN, USA) or an Atricure clamp (Atricure, West Chester, OH, USA). After the aorta clamping and cardioplegia perfusion, incising the LA and performing the LA ablation lines, which included bilateral pulmonary vein isolations (PVI), a roof connecting line between both islands of PVIs and a line from the left PVI ablation lesion to the posterior mitral annulus. Then, LAA closure ablation was performed by making a radiofrequency lesion around the base of LAA. Patients who underwent LAA interventions experienced either LAA resection or LAA endocardial closure. The LAA was excised with surgical staplers (Covidien, Medtronic, Minneapolis, MN, USA). Whether to perform LAA interventions or preservations depended on several factors, which included the anatomical features of LAA, the risk of atrial rupture evaluated by surgeons, the risk of stroke and surgeons’ preferences. All patients experienced the division of the Marshall ligament. The right atrial ablation lesions included the cavotricuspid isthmus ablation, superior vena cava to inferior vena cava, lateral free-wall to anterior-medial tricuspid valve annulus; and medial free-wall to the anterior-medial tricuspid valve annulus. More details about the above procedures can be found in our **Supplementary Material**.

### 2.4 Postoperative Medical Treatments

As we described in the prior study [14], oral anticoagulation was recommended to everyone for the first three months after procedures. For patients who had LAA preserved, oral anticoagulation was discontinued if one had no AF recurrence and a CHADS2 score <two within three months following the procedure. All patients who underwent LAA treatments were recommended to discontinue oral anticoagulation 3 months following the procedure. Unless contraindicated, patients received amiodarone or sotalol within 24 h of the procedure, which was discontinued at three months (blanking period). Antiarrhythmic drugs (AAds) were maintained or restarted if patients demonstrated recurrent AF. Additional postoperative medications were shown in **Supplementary Material**. Multiple agents, including beta-blockers, nondihydropyridine calcium-channel blockers (CCBs), digoxin and certain AADs were used for rate control in patients with AF persistence. Excluding the above treatments, angiotensin-converting enzyme inhibitors (ACEI), angiotensin receptor blockers (ARB), aldosterone receptor antagonists (ARA), and loop diuretics were used to relieve symptoms and delay the further exacerbation of MR during follow-up according to the patient’s degree of symptoms, grade of MR, hemodynamic situations, and presence or absence of heart failure.

### 2.5 Outcomes during Follow-Up

Clinical visits were arranged for all patients at 1, 3, and 12 months after procedures, and then annually thereafter. Each clinical visit was composed of medical interrogation, physical examination, X-ray, Holter examination, and echocardiographic evaluation. The primary outcome was defined as the worsening of MR (MR grade increased from mild to moderate, mild to severe or moderate to severe). Adverse clinical events including all-cause death, recurrent atrial fibrillation and stroke were recorded. Excluding the routine echocardiographic parameters, items related to the anatomy of the mitral valve including MAD and tethering height (TH) were also recorded at each visit.

### 2.6 Statistic

Continuous variables which fit the normal distribution were presented as mean (standard deviation), while other continuous variables were presented as median (interquartile range). Categorical values were presented as percentages, and odds ratios (OR) were presented with 95% confidence intervals (CIs). Unadjusted comparisons in terms of categorical variables between different cohorts were done by the chi-square test and Fisher’s test. Unadjusted comparisons in terms of continuous variables between different cohorts were done by the two-sample *t* test and Kruskal-Wallis H test. Propensity scores in this model were acquired through the generalized boosted model. The absolute standardized mean difference (ASMD) was used to measure the difference between two univariate distributions of a single baseline variable [15]. Imbalance was presented when a value was ≥0.10. Freedom from outcomes whose results were adjusted by inverse probability of treatment weighting (IPTW) for casual effects after acquiring propensity scores. All significance tests were 2-tailed, and a *p* value of <0.05 was considered to be statistically significant. R version 3.6.2 (R Foundation for Statistical Computing, Vienna, Austria) was used for all statistical analysis.

## 3. Results

### 3.1 Baseline Characteristics

A total of 456 patients were enrolled in this study. Of these, 278 underwent LAA interventions (146 cases of resection, 132 cases of endocardial closure), and the remaining 177 had LAA preserved. The average age was 62 ± 7.6 years; male patients accounted for 41.4% of the overall population. Before ablations, patients with mild to moderate MR (Grade II) accounted for 95.8% of the overall population, and the remaining patients showed moderate to severe (Grade III) MR. After IPTW adjustments, no significant differences were observed among the three groups. All ASMDs were also decreased to lower than 10% after adjustments (Table 1).

**Table 1.** **Baseline characteristics**.

Characteristics		LAA excision	LAA closure	LAA preservation	Unadjusted maximal ASMD (%)	IPTW-Adjusted maximal ASMD (%)
		(n = 146)	(n = 132)	(n = 178)		
Male, no. (%)		53 (36.3)	65 (49.2) *	69 (38.8) ^†^	26.3	9.5
Age (years), mean (SD)		62.1 (6.5)	61.1 (7.4)	62.1 (8.5)	12.1	6.4
BMI, mean (SD)		23.6 (3.7)	24.3 (3.3)	23.7 (3.7)	20.2	6.8
NYHA, no. (%)						
	II	130 (89.0)	118 (89.4)	157 (88.2)	2.7	3.5
	III	16 (11.0)	14 (10.6)	21 (11.8)	2.7	3.5
Hypertension, no. (%)		50 (34.2)	52 (39.4)	52 (29.2)	21.5	9.1
Diabetes, no. (%)		20 (13.7)	18 (13.6)	23 (12.9)	2.3	4.8
HLP, no. (%)		32 (21.9)	27 (20.5)	42 (23.6)	7.6	8.2
Smoke, no. (%)		21 (14.4)	27 (20.5)	37 (20.8)	16.4	2.6
Alcohol, no. (%)		22 (15.1)	25 (18.9)	37 (20.8)	14.7	4.1
Stroke, no. (%)		17 (11.6)	17 (12.9)	23 (12.9)	3.9	8.8
Thyroid dysfunction, no. (%)		5 (3.4)	2 (1.5)	4 (2.2)	12.4	5.6
PMI, no. (%)		7 (4.8)	8 (6.1)	14 (7.9)	12.6	4.7
HF, no. (%)		10 (6.8)	6 (4.5)	17 (9.6)	19.4	1.8
COPD, no. (%)		6 (4.1)	5 (3.8)	7 (3.9)	1.7	0.7
CKD, no. (%)		2 (1.4)	4 (3.0)	4 (2.2)	11.3	4.9
CHA2DS2-VASC score						
	0–1	47 (32.2)	49 (37.1)	100 (56.2)	1.6	9.4
	≥2	99 (67.8)	81 (61.4)	78 (43.8)	1.6	9.4
Ablation history, no. (%)		10 (6.8)	13 (9.8)	12 (6.7)	11.7	9.2
LVEDD (mm), mean (SD)		45.6 (4.6)	45.9 (4.5)	46.1 (4.6)	10.6	1.5
LVESD (mm), mean (SD)		31.8 (5.5)	31.5 (5.7)	31.6 (5.1)	4.9	6.8
LVEF (%), mean (SD)		61.4 (5.3)	61.7 (6.0)	61.8 (7.2)	6.4	2.0
LAD (mm), mean (SD)		46.9 (6.1)	45.9 (6.5) *	46.0 (6.8)	24.6	3.1
MAD (mm), mean (SD)		31.5 (3.4)	31.4 (2.7)	31.5 (2.6)	1.6	6.2
MR grade, no. (%)						
	+∼++	129 (88.4)	130 (98.5) *	178 (100) *	16.3	6.6
	+++	17 (11.6)	2 (1.5) *	0 (0) *	16.3	6.6
TR grade, no. (%)						
	Trivial or mild	87 (59.6)	82 (62.1)	97 (50.5) ^†^	7.7	5.1
	Moderate	57 (39.0)	50 (37.9)	77 (43.2)	7.7	5.1
	Severe	2 (1.4)	0 (0)	4 (2.2)	7.7	5.1

SD, standard deviation; LAA, left atrial appendage; BMI, body mass index; NYHA, New York Heart Association; HLP, hyperlipemia; PMI, post myocardial infarction; HF, heart failure; COPD, chronic obstructive pulmonary disease; CKD, chronic kidney disease; LVEDD, left ventricular end diastolic diameter; LVESD, left ventricular end systolic diameter; LVEF, left ventricular ejection fraction; LAD, left atrial diameter; MAD, mitral annular diameter; MR, mitral regurgitation; TR, tricuspid regurgitation; IPTW, inverse probability of treatment weighting; ASMD, absolute standardized mean difference. *p*-values may not be interpreted as confirmatory but rather descriptive. *p < 0.05 *vs*. LAA excision; ^†^*p* < 0.05 *vs*. LAA closure.

### 3.2 Survival Outcomes

**Fig. 1.**
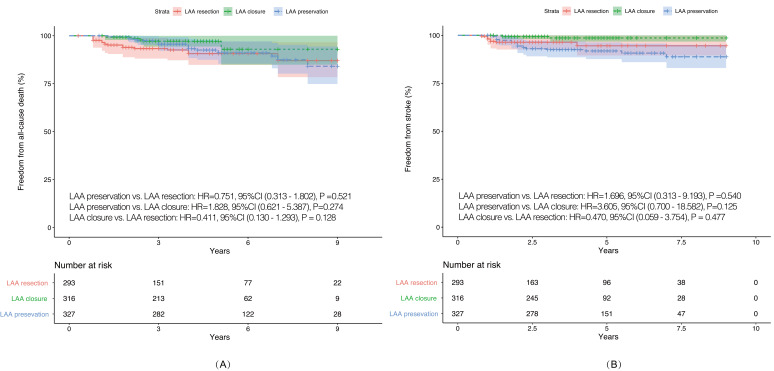
**IPTW adjusted Kaplan-Meier Curves in terms of Mortality and Stroke**. (A) IPTW adjusted Kaplan-Meier curves in terms of all-cause mortality. (B) IPTW adjusted Kaplan-Meier curves in terms of stroke. IPTW, inverse probability of treatment weighting; LAA, left atrial appendage; HR, hazard ratio; CI, confidence interval.

All patients in this study successfully underwent the ablation procedures, and no in-hospital mortalities were observed. After a median follow-up time of 4 years, 30 deaths were observed. The follow-up rate of this study was 98.5%. The overall survival rates at 3 years and 5 years were 94.2% and 89.1%, respectively. No differences were observed among the three groups after IPTW adjustment (All *p* > 0.05) (Fig. 1A). During follow-up, a total of 11 cases of stroke events were observed. The differences in terms of the incidence of stroke were also not significant between LAA intervention and LAA preservation (Fig. 1B).

### 3.3 Mitral Regurgitations and Rhythm Status during Follow-Up

**Fig. 2.**
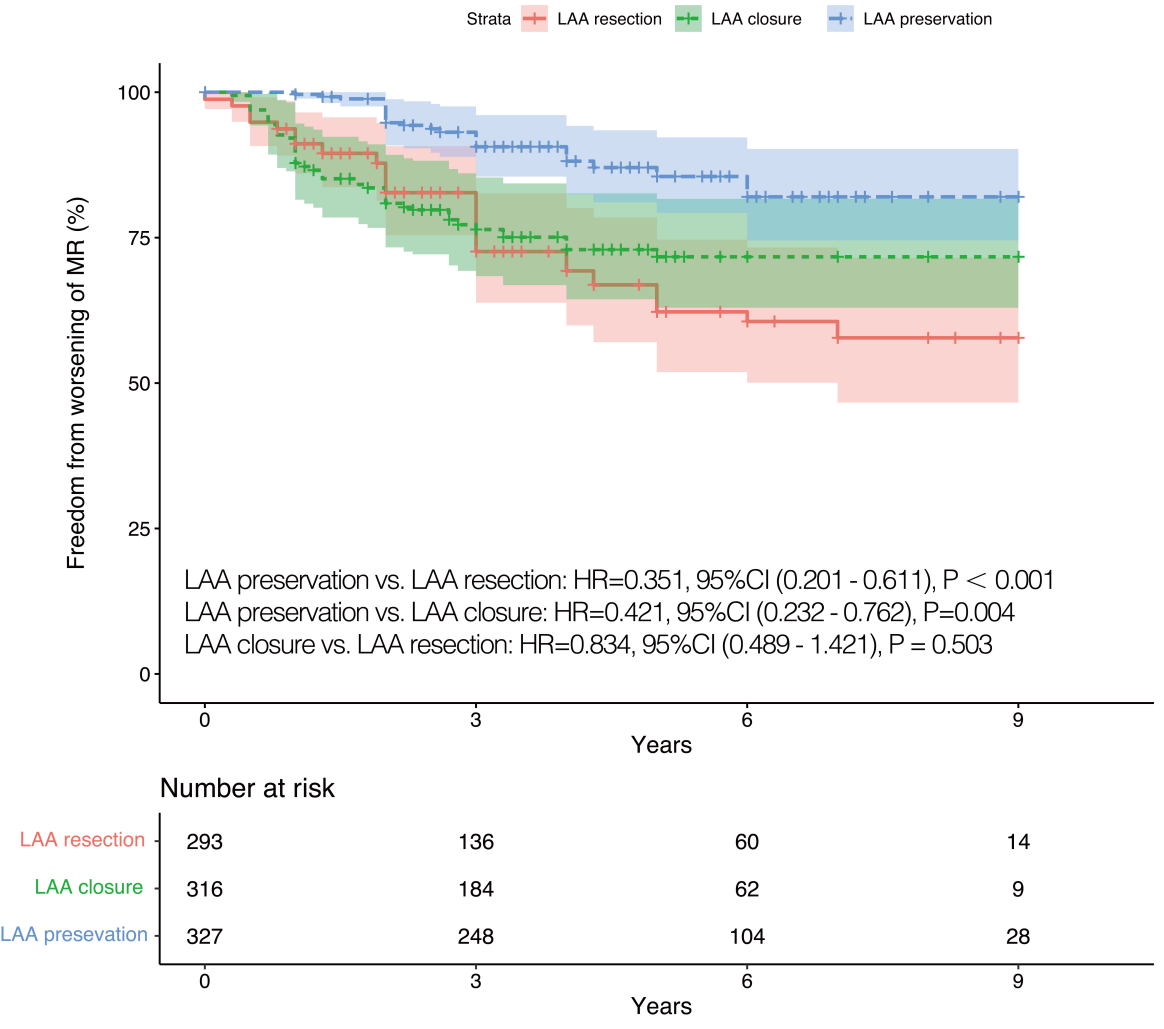
**IPTW adjusted Kaplan-Meier curves in terms of worsening of MR. **IPTW, inverse probability of treatment weighting; LAA, left atrial appendage; HR, hazard ratio; CI, confidence interval; MR, mitral regurgitation.

**Fig. 3.**
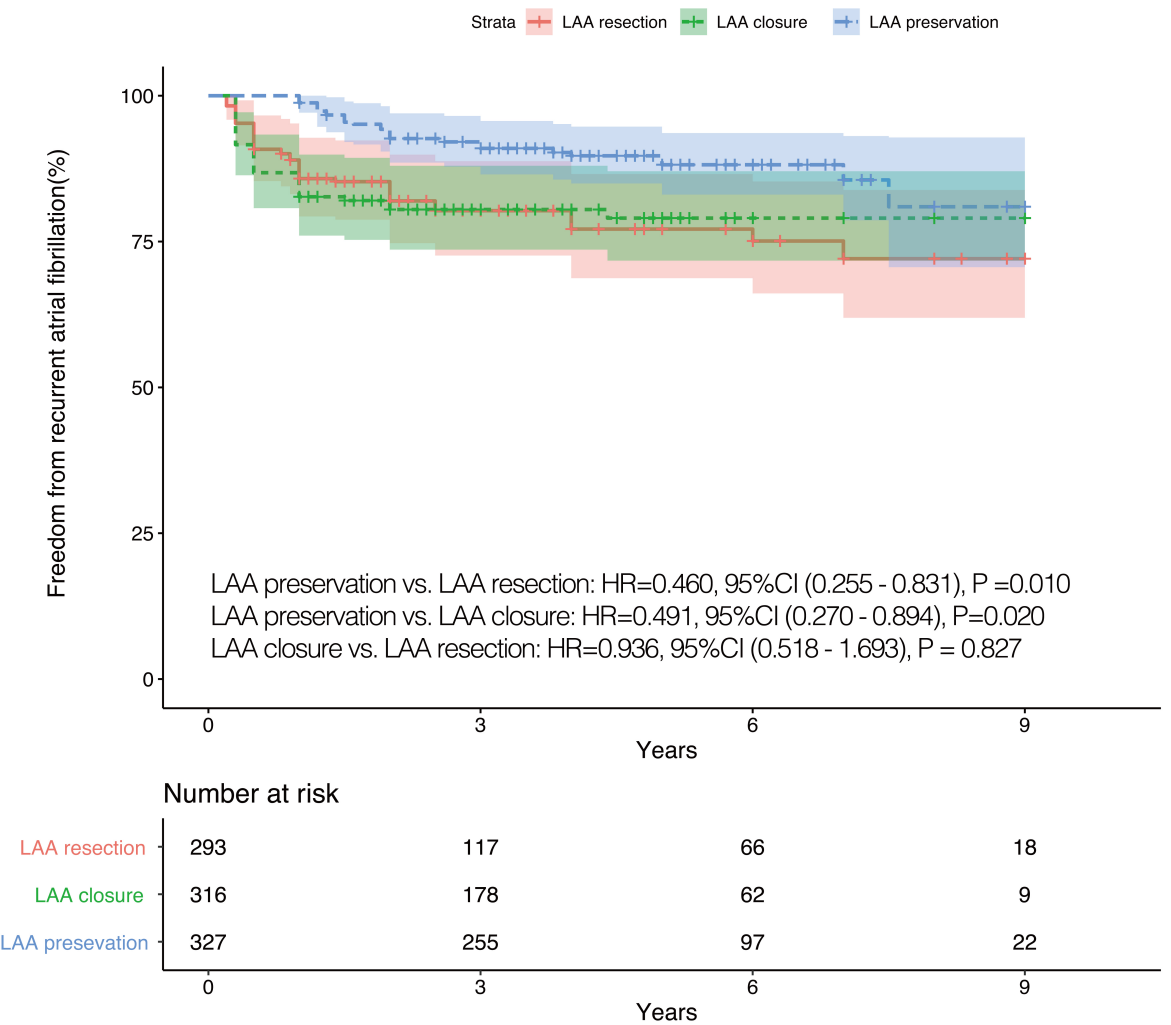
**IPTW adjusted Kaplan-Meier curves in terms of recurrent AF**. IPTW, inverse probability of treatment weighting; LAA, left atrial appendage; HR, hazard ratio; CI, confidence interval; AF, atrial fibrillation.

As shown in Table 2, medications after discharges showed no differences among the three groups. Worsening of MR occurred in 20.2% (92 cases) of participants during follow-up. Of these, 52 cases of MR aggravated from mild to moderate, 10 cases aggravated from mild to severe, and 30 cases aggravated from moderate to severe. Worsening of MR was more likely to occur among patients who underwent LAA resected or closed (LAA preservation *vs*. LAA endocardial closure: *p* = 0.004, 95% CI (0.232–0.762); LAA preservation *vs*. LAA resection: *p* < 0.001, 95% CI (0.201–0.611)) (Fig. 2). Patients who had LAA preserved had a lower risk of recurrent AF than those who underwent LAA interventions (LAA preservation *vs*. LAA endocardial closure: *p* = 0.020, 95% CI (0.270–0.894); LAA preservation *vs*. LAA resection: *p* = 0.010, 95% CI (0.255-0.831)) (Fig. 3). All results without IPTW adjustments can be found in the **Supplementary Material**.

**Table 2.** **Medications after discharges**.

	LAA excision	LAA closure	LAA preservation	*p* value
	(n = 146)	(n = 132)	(n = 178)	
AADs, no. (%)	72 (49.3)	61 (46.2)	87 (48.9)	0.855
β-blocker, no. (%)	86 (58.9)	83 (62.9)	96 (53.9)	0.280
Oral anticoagulant, no. (%)	41 (28.1)	38 (28.9)	44 (24.7)	0.680
Loop-diuretic, no. (%)	51 (34.9)	50 (37.9)	66 (37.1)	0.867
ARA, no. (%)	32 (21.9)	24 (18.2)	35 (19.7)	0.733
ACEI, no. (%)	28 (19.2)	22 (16.7)	33 (18.5)	0.854

Any changes in medications for the worsening of MR were not included in this table. AADs, anti-arrhythmia drugs; ACEI, angiotensin-converting enzyme inhibitors; ARA, aldosterone receptor antagonist; LAA, left atrial appendage; MR, mitral regurgitation. *p*-values may not be interpreted as confirmatory but rather descriptive.

### 3.4 Longitudinal Changes in Echocardiographic Estimates

**Fig. 4.**
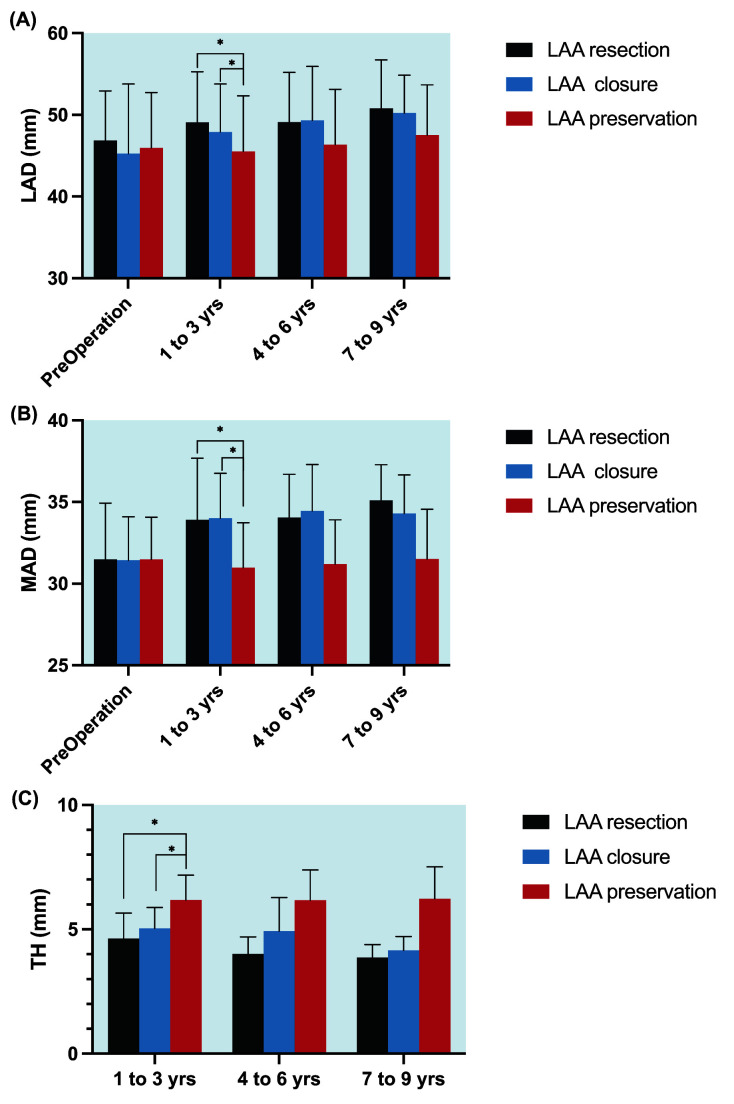
**Longitudinal echocardiographic features during follow-up among different LAA treatment methods**. (A) LAD among different LAA treatment methods; (B) MAD among different LAA treatment methods; (C) TH among different LAA treatment methods; *p < 0.05; The errors bars are SE of the mean. No corrections for multiple testing were applied. LAA, left atrial appendage; LAD, left atrial diameter; MAD, mitral annular diameter; TH, tenting height; *p*-values may not be interpreted as confirmatory but rather descriptive.

Before ablations, the three groups of patients showed similar left atrial diameter (LAD) and MAD (all *p* > 0.05). By 3 years postoperatively, patients who underwent LAA resection and endocardial closure showed significantly larger LAD and MAD than those who had LAA preserved (*p* < 0.05). At any time of follow-up (1 to 3 years, 4 to 6 years, and 7 to 9 years postoperatively), patients who underwent LAA interventions (resection or endocardial closure) showed lower TH than those who had LAA preserved (Fig. 4).

## 4. Discussion

This article is the first study to investigate the impacts of different LAA treatments on patients with mitral diseases. We found that patients who underwent LAA resection or endocardial closure during surgical ablations had a higher risk of MR worsening than those who had LAA preserved. A worse coaptation of the mitral valve may be present among those who lost LAA, which may contribute to the worsening of MR.

The LAA derives from the primordial LA, which is a finger-like projection from the main body of LA [2]. The mechanical and endocrinological functions of LAA are hard to ignore [3]. LAA plays an important role in modulating the LA pressure through its distensibility. In addition, the concentration of atrial natriuretic peptide (ANP) is the largest in LAA, which could also help to modulate the LA pressure [1]. In 1990, Davis *et al*. [16] first reported that the slope of the LA pressure *vs*. normalized volume data increased significantly when the LAA was excluded. Recently, more evidence showed left atrial enlargement or left atrial remodeling was present after LAA interventions [9, 10, 17], which may be related to the postoperative decreases of ANP [18]. In the study of Kim *et al*. [8], they found postoperative LA transport functions were more favorable with LAA preservation than with LAA interventions among patients who underwent surgical ablations. The loss of left atrial physiological function may explain why patients who underwent LAA interventions showed larger LAD and MAD during follow-up in our study.

Patients with mild or moderate MR were commonly not considered as candidates for invasive MV interventions [19, 20]. In our study, the enlargements of LA and MV annulus were more frequently observed among patients who underwent LAA interventions, which was in accordance with the results from the above studies. Despite both enlargements of LA and MV annulus, one of the most symbolic characteristics of MR among patients who underwent LAA interventions is the shortening of MV tenting height [11]. During follow-up, the mean TH of patients who underwent LAA resections and endocardial closure were 4.63 mm and 5.04 mm, respectively, which were significantly lower than the value of the normal population in the study Ring *et al*. [21]. All the above evidence showed that LAA interventions could affect patients’ clinical outcomes by modulating LA functions.

Additionally, some relationships may be present between LAA interventions and recurrent AF. In the study of Melduni *et al*. [6], surgical LAA closure during routine non-AF-related cardiac surgery was independently associated with an increased risk of early postoperative AF. Similarly, patients who underwent LAA interventions in our study were at a higher risk of recurrent AF during follow-up, which may be caused by the increased pressure and decreased distensibility of LA.

This is a single-center, retrospective study, all baseline clinical features, rhythm status and MR status were retrospectively collected. Our study has the typical limitations of retrospective analysis. Additionally, the baseline tenting heights of different groups were lacking, because tenting height itself was not a common examination item in our center, we only acquired it in the follow-up echo.

## 5. Conclusions

Our findings further confirmed the regulating function of LAA, which could affect LA remodeling. Mitral regurgitation was more likely to get worse when patients with fundamental mitral diseases underwent LAA closure during isolated surgical AF ablations.

In the absence of LAA, dilation of the left atrium and the mitral annulus may lead to a reduction of the coaptation area, ultimately causing increased regurgitation.

## Data Availability

The datasets used and/or analyzed during the current study are available from the corresponding author upon reasonable request.

## Supplementary Material

Supplementary material associated with this article can be found, in the online version, at https://doi.org/10.31083/j.rcm2501013.

## References

[1] Delgado V, Di Biase L, Leung M, Romero J, Tops LF, Casadei B (2017). Structure and Function of the Left Atrium and Left Atrial Appendage. *Journal of the American College of Cardiology*.

[2] Di Biase L, Burkhardt JD, Mohanty P, Sanchez J, Mohanty S, Horton R (2010). Left Atrial Appendage. *Circulation*.

[3] Yaghi S, Song C, Gray WA, Furie KL, Elkind MSV, Kamel H (2015). Left Atrial Appendage Function and Stroke Risk. *Stroke*.

[4] January CT, Wann LS, Calkins H, Chen LY, Cigarroa JE, Cleveland JC (2019). 2019 AHA/ACC/HRS Focused Update of the 2014 AHA/ACC/HRS Guideline for the Management of Patients With Atrial Fibrillation: A Report of the American College of Cardiology/American Heart Association Task Force on Clinical Practice Guidelines and the Heart Rhythm Society in Collaboration With the Society of Thoracic Surgeons. *Circulation*.

[5] Nishimura M, Lupercio-Lopez F, Hsu JC (2019). Left Atrial Appendage Electrical Isolation as a Target in Atrial Fibrillation. *JACC: Clinical Electrophysiology*.

[6] Melduni RM, Schaff HV, Lee H, Gersh BJ, Noseworthy PA, Bailey KR (2017). Impact of Left Atrial Appendage Closure during Cardiac Surgery on the Occurrence of Early Postoperative Atrial Fibrillation, Stroke, and Mortality. *Circulation*.

[7] Mahmood E, Matyal R, Mahmood F, Xu X, Sharkey A, Chaudhary O (2020). Impact of Left Atrial Appendage Exclusion on Short-Term Outcomes in Isolated Coronary Artery Bypass Graft Surgery. *Circulation*.

[8] Kim HJ, Chang D, Kim S, Kim JK, Kim K, Jung S (2022). Left atrial appendage preservation versus closure during surgical ablation of atrial fibrillation. *Heart*.

[9] Luani B, Rauwolf T, Groscheck T, Tanev I, Herold J, Isermann B (2018). Serial Assessment of Natriuretic Peptides in Patients Undergoing Interventional Closure of the Left Atrial Appendage. *Heart, Lung and Circulation*.

[10] Luani B, Groscheck T, Genz C, Tanev I, Rauwolf T, Herold J (2017). Left atrial enlargement and clinical considerations in patients with or without a residual interatrial shunt after closure of the left atrial appendage with the WATCHMAN™-device. *BMC Cardiovascular Disorders*.

[11] Deferm S, Bertrand PB, Verbrugge FH, Verhaert D, Rega F, Thomas JD (2019). Atrial Functional Mitral Regurgitation. *Journal of the American College of Cardiology*.

[12] Hoit BD (2020). Atrial functional mitral regurgitation. *Current Opinion in Cardiology*.

[13] Zoghbi WA, Adams D, Bonow RO, Enriquez-Sarano M, Foster E, Grayburn PA (2017). Recommendations for Noninvasive Evaluation of Native Valvular Regurgitation: A Report from the American Society of Echocardiography Developed in Collaboration with the Society for Cardiovascular Magnetic Resonance. *Journal of the American Society of Echocardiography*.

[14] Wang J, Li S, Ye Q, Ma X, Zhao Y, Han J (2020). Catheter ablation or surgical therapy in moderate-severe tricuspid regurgitation caused by long-standing persistent atrial fibrillation. Propensity score analysis. *Journal of cardiothoracic surgery*.

[15] McCaffrey DF, Griffin BA, Almirall D, Slaughter ME, Ramchand R, Burgette LF (2013). A tutorial on propensity score estimation for multiple treatments using generalized boosted models. *Statistics in Medicine*.

[16] Davis CA, Rembert JC, Greenfield JC (1990). Compliance of left atrium with and without left atrium appendage. *American Journal of Physiology-Heart and Circulatory Physiology*.

[17] Yamanaka K, Sekine Y, Nonaka M, Iwakura A, Yoshitani K, Nakagawa Y (2010). Left atrial appendage contributes to left atrial booster function after the maze procedure: quantitative assessment with multidetector computed tomography. *European Journal of Cardio-Thoracic Surgery*.

[18] Cruz-Gonzalez I, Palazuelos Molinero J, Valenzuela M, Rada I, Perez-Rivera JA, Arribas Jimenez A (2016). Brain natriuretic peptide levels variation after left atrial appendage occlusion. *Catheterization and Cardiovascular Interventions*.

[19] Otto CM, Nishimura RA, Bonow RO, Carabello BA, Erwin JP, Writing Committee Members (2021). 2020 ACC/AHA Guideline for the Management of Patients With Valvular Heart Disease: Executive Summary: A Report of the American College of Cardiology/American Heart Association Joint Committee on Clinical Practice Guidelines. *Journal of the American College of Cardiology*.

[20] Baumgartner H, Falk V, Bax JJ, De Bonis M, Hamm C, Holm PJ (2017). 2017 ESC/EACTS Guidelines for the management of valvular heart disease. *European Heart Journal*.

[21] Ring L, Dutka DP, Wells FC, Fynn SP, Shapiro LM, Rana BS (2014). Mechanisms of atrial mitral regurgitation: insights using 3D transoesophageal echo. *European Heart Journal Cardiovascular Imaging*.

