# Supplementary material for: Impacts of Left Atrial Appendage Treatments on Mitral Valve Diseases during Surgical Ablations
Source: Rev Cardiovasc Med. 2024 Jan 9;25(1):13. doi: 10.31083/j.rcm2501013 (PMC11262407; doi:10.31083/j.rcm2501013)
Supplement: Supplementary file 1 [file 2153-8174-25-1-013-s1.docx]

**Unadjusted Kaplan-Meier Curve (Before IPTW)**

***(1) Kaplan-Meier curve in terms of all-cause death***


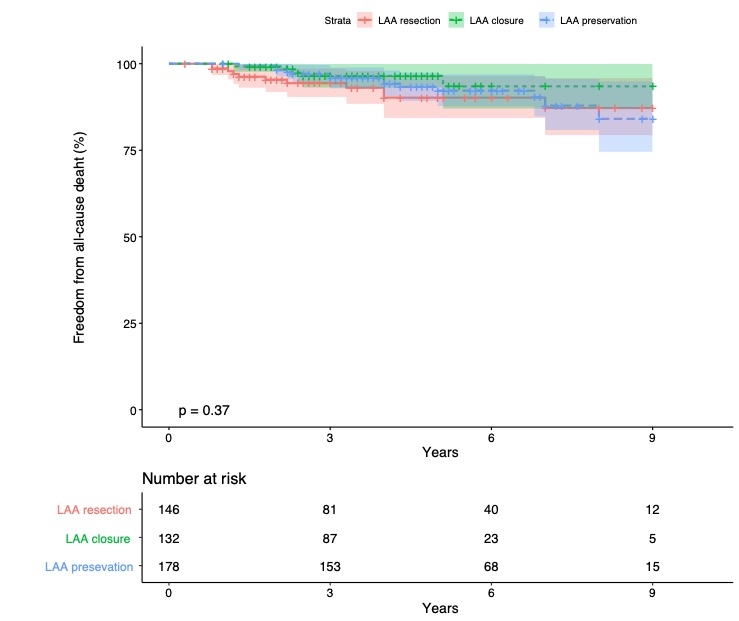


**LAA, left atrial appendage.**

***(2) Kaplan-Meier curve in terms of stroke***


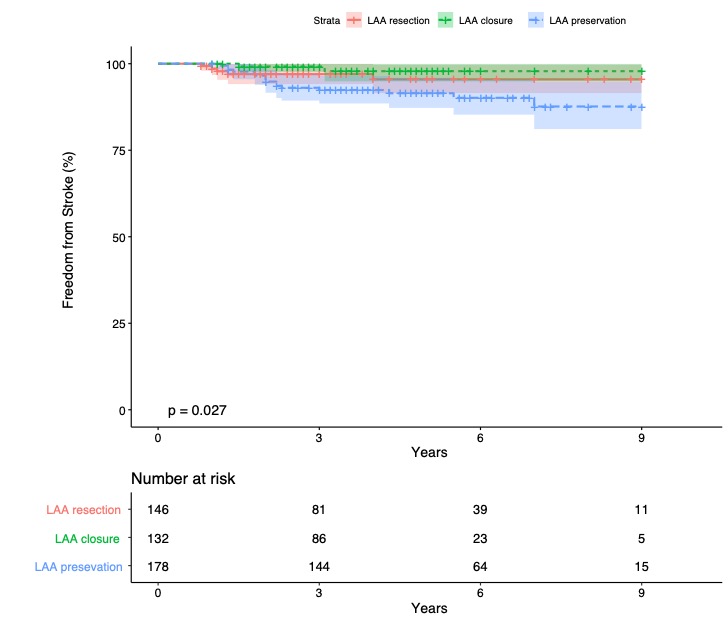


**LAA, left atrial appendage.**

***(3) Kaplan-Meier curve in terms of aggravation of MR***


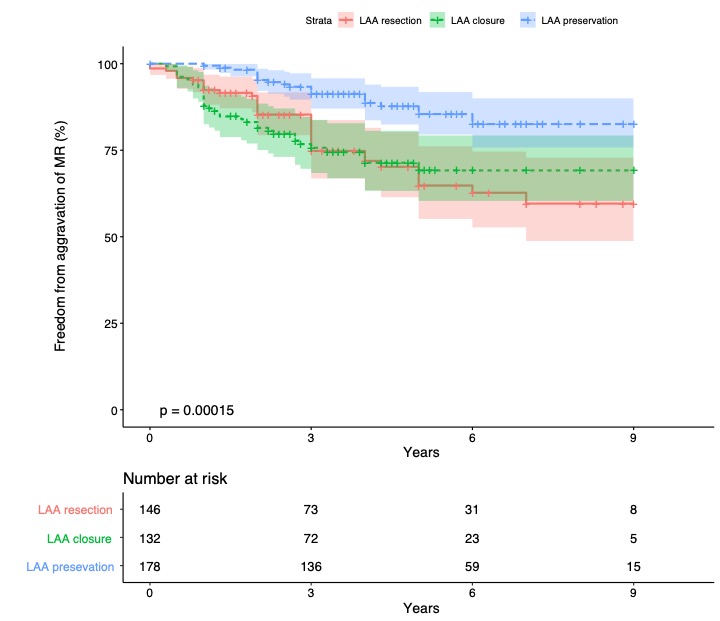


**LAA, left atrial appendage; MR, mitral regurgitation**

***(4) Kaplan-Meier curve in terms of aggravation of recurrent AF***

***
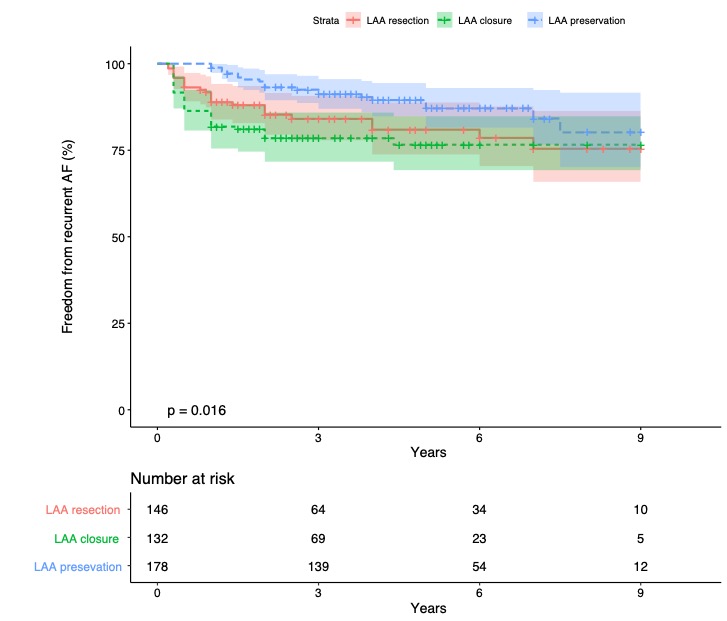
***

**LAA, left atrial appendage; AF, atrial fibrillation**

**Ablation Strategy.**


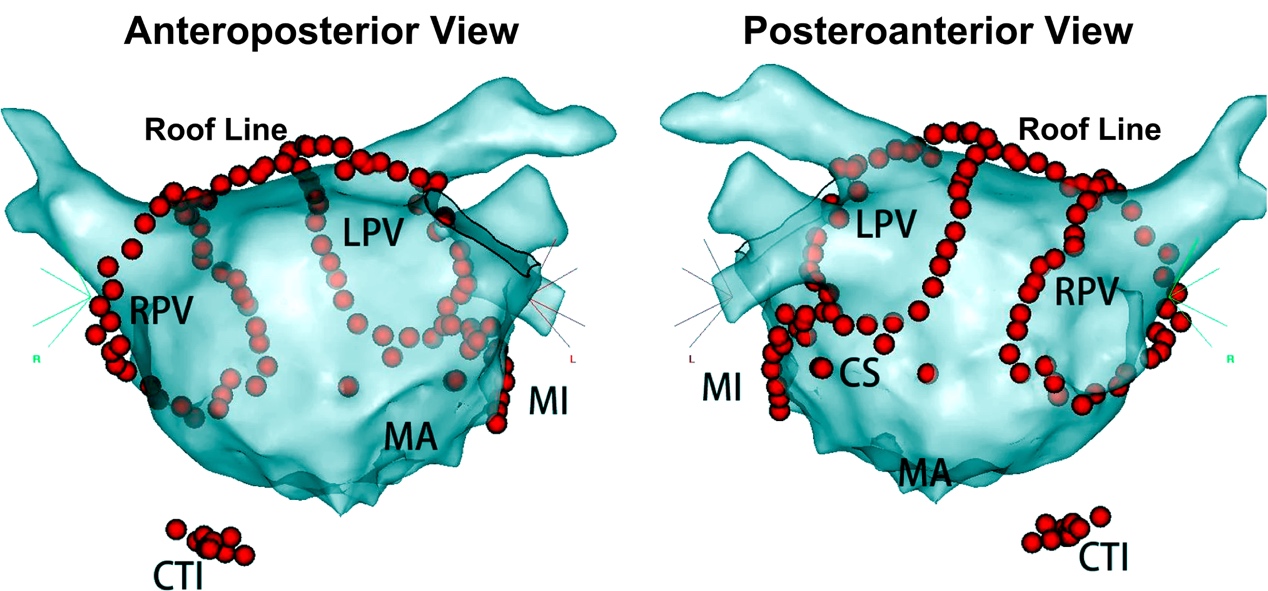


**The red dots represented endocardial ablation. CS, coronary sinus; CTI, cavotricuspid isthmus; LPV, left pulmonary vein; MA, mitral annulus; MI, mitral isthmus; RPV, right pulmonary vein**

**Left atrial appendage treatments.**

**
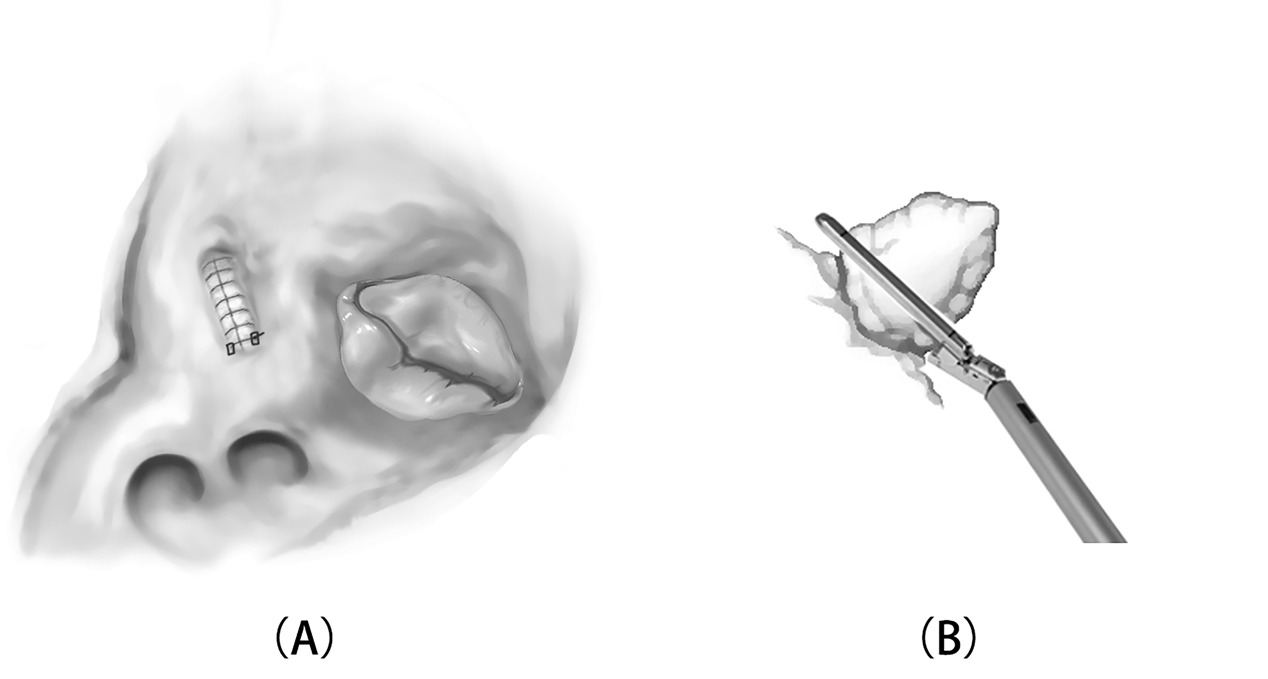
**

**(A) Left atrial appendage endocardial closure under direct vision; (B) Left atrial appendage excision by stapler.**
